# Rescue of caprine fetal ovaries, vitrification and follicular development after xenotransplantation in two immunodeficient mice models

**DOI:** 10.1590/1984-3143-AR2019-0115

**Published:** 2020-06-29

**Authors:** Muriel Magda Lustosa Pimentel, Fernanda Araujo dos Santos, Luã Barbalho de Macêdo, Parmênedes Dias de Brito, Gabriela Liberalino Lima, Raimundo Alves Barreto, Marcelo Barbosa Bezerra

**Affiliations:** 1 Centro Universitário Cesmac, Maceió, AL, Brasil; 2 Programa de Pós-graduação em Ciência Animal, Universidade Federal Rural do Semi-Árido, Mossoró, RN, Brasil; 3 Departamento de Ciência Animal, Instituto Federal de Educação, Ciência e Tecnologia do Ceará, Crato, CE, Brasil; 4 Departamento de Ciência Animal, Universidade Federal Rural do Semi-Árido, Mossoró, RN, Brasil

**Keywords:** cryopreservation, assisted reproductive technology, livestock

## Abstract

Domestic and wild goats are very susceptible animals to predation, specially when pregnancy occurs. This study aimed to evaluate the use of goat fetal ovarian tissue for vitrification followed by xenotransplantation and fresh xenotransplantation in two immunosuppressed mice models (C57BL/6 SCID and Balb-C NUDE). Goat fetus ovaries were collected in slaughterhouses, divided into small cortical pieces and were destined for fresh xenotransplantation (FX) and cryopreservation followed by xenotransplantation (CX). Five recipients from each lineage were used for FX and 10 animals from each lineage for CX. The mice were euthanized after 65 postoperative days, and the transplants were collected for microscopic assessment. The blood plasma was collected for estradiol measurement. Independently of mice strain, all recipients presented complete estrus cycle in FX and 80% after CX groups. Follicles were observed at all development stages without morphological changes. The volume density and total vessel surface observed in the transplants were different (p <0.01) between groups. The estradiol levels in the recipients did not differ (p <0.05) among the treatments. Thus, it is possible to activate the preantral follicles in the ovaries of fetuses by optimizing germplasm utilization and conservation of domestic and endangered wild goats that are in predatory situations, undesirable drowning or accidental death, since provided conditions for xenotransplantation are performed.

## Introduction

In recent years, a growing number of studies have been associating goat species with commercially established reproductive biotechniques, aiming to increase the productivity of these animals in breeding programs. Among the mentioned biotechnology, we highlight Artificial Insemination (Pellicer-Rubio et al., 2016), Embryo Transfer (Fonseca et al., 2016) and *in vitro* Production of Embryos (Hajian et al., 2017). Goats have also been the species of choice in developing techniques such as Cloning (Yang et al., 2016), Transgenesis (Bertolini et al., 2016) and as an association model between both using goat fetuses as donors (Baguisi et al., 1999). The development of preantral follicles have been experimentally studied in vitro (Saraiva et al., 2010; Magalhães et al., 2011; Leal et al., 2018) and *in vivo* by Autotransplantation (Santos et al., 2009, Donfack et al., 2018). Additionally, these animals can be used as experimental models for humans, furnishing important results in endocrinology and for assessment of ovarian function, corroborating the observations of physiological similarities between humans and goats in these processes (Liu et al., 2008).

In another context, the incidence of small wild ruminants of autochthonous breeds or imported for commercial purposes, that suffer predation is a recurrent fact in the nature and in small livestock farms. Specifically, domestic and wild goats are easy prey for domestic and wild carnivores (Durawo et al., 2017; Roshnath et al., 2017; Khan et al., 2018), and once pregnant, newborn and kids of goats exhibit less mobility and are more easily predated (Durawo et al., 2017), the loss of genetic material in these conditions of vulnerability may prove invaluable.

Based on the context described above, researches have been carried out in the animal reproduction area aiming to increase the reproductive potential of animals with high zootechnical value (Commin et al., 2012) or preservation of endangered mammals (Gastal et al., 2017) such as cervids (Comizzoli and Wildt, 2013; Comizzoli, 2015). Ovary tissue xenotransplantation is a potential technique for maintaining and developing genetic material, especially when combined with germplasm preservation methods such as vitrification, allowing better planning and preparation by the involved research teams. In addition, the combination of ovarian tissue cryopreservation with xenotransplantation in immunodeficient recipients has demonstrated scientific relevance with considerable follicular development (Santos et al., 2010; Aerts et al., 2010; Wiedemann et al., 2012; Praxedes et al., 2018).

Aiming to reduce rejections after xenotransplantation, genetic manipulations were carried out by researchers who produced immunodeficient rodents to be used as recipients, for example the C57BL6 SCID and BALB / c Nude lines, with the possibility of using both female (Senbon et al., 2005) and males receptors (Hernandez-Fonseca et al., 2005) for the performance of ovarian xenotransplantation. This being an indispensable requirement for the development of transplanted tissues. Thus, this study aimed to evaluate the development and viability of fresh xenotransplantation or xenotransplantation after cryopreservation of fetal goat ovarian tissue in two different immunodeficient mice lineages.

## Material and methods

This work was carried out in accordance with the recommendations of the Brazilian Code of Animal Experimentation (COBEA) (1988) and was approved by the Ethics Committee on Animal Use (CEUA) of the institution under protocol number 23091006934/2015-73.

### Ovarian donors

Ovaries from goat fetuses aged 120 to 140 days of gestation were collected immediately after slaughtering pregnant females at the local slaughterhouse. The ovaries were washed with 0.9% saline solution, inserted in sterile conical tubes (50 mL), identified according to the slaughter sequence, and then transported in saline with antibiotics (100 μg/mL of penicillin) and kept at a temperature of 37º until arriving at the laboratory to be processed.

Six pairs of goat fetus ovaries with gestational age ranging from 120 to 140 days, undefined breed, were collected from the local slaughterhouse. The ovaries were washed with 0.9% saline solution, then transported in sterile tubes (50 mL) containing 0.9% saline solution with 100 μg/mL penicillin at room temperature.

Ovarian cortical tissue was identified and divided into small pieces of approximately 1 mm^3^ which were then separated into three groups: control (prior transplantation for histological analysis), freshly transplanted (FX): transplanted without vascular reanastomosis under the renal capsule of the kidney (five fragments per recipient), and cryopreserved (CX) (also transplanted after 15 days of cryopreservation). The fragments were kept in Minimum Essential Medium (MEM) until the time of transplantation, not exceeding the five-hour time limit from harvest or thawing to transplantation.

### Cryopreservation of cortical pieces

The pooled cortical pieces were used for the cryopreserved group. The vitrification solution (VS) consisted of 3 mols/L dimethyl sulfoxide (DMSO) in Minimum Essential Medium (MEM) supplemented with 0.25 M sucrose (SUC) and 10% fetal calf serum (FCS). After that moment, the samples were exposed in 1.8 mL of VS for 5 minutes and the solution excess was withdrawn with sterile gauze, placed on a metal bucket surface partially immersed in liquid nitrogen (LN_2_) and then vitrified. After that, the sample was transferred (with nitrogen-cooled clamp) to cryotubes for storage in LN_2_ (-196 °C) (Carvalho et al., 2011).

After 2 weeks the samples were thawed at room temperature (approximately 25 °C) for 1 min and then immersed in a 37 °C water bath for 5 s. DMSO was removed from the ovarian fragment by three consecutive washes for 5 min in MEM supplemented with 10% FCS and decreasing sucrose concentrations (0.50, 0.25 and 0 mol/L). Finally, the cortical pieces were xenotransplanted in the recipients (Praxedes et al., 2018).

### Mice recipients

Immunodeficient mice of the C57BL/6 SCID (n = 15) and Balb-C NUDE (n = 15) lineages, ageing between 20-24 weeks were housed in sterile mini-cages and maintained under a light/dark cycle of 12 hours at 22 °C and fed ad libitum. For surgical procedures 5 animals/lineage were used to perform the fresh xenotransplantation (FX) and 10 animals/lineage for cryopreserved xenotransplantation (CX).

### Xenotransplantation procedure

Recipients were anesthetized with 2.5% 2,2,2-tribromoethanol at the dose of 18 ml kg^-1^ (SCID) and 20 ml kg^-1^ (NUDE) by intraperitoneal administration. Dosages were established from previous experiments (Pimentel et al., 2017).

After bilateral ovariectomy, five pieces of the pooled ovarian tissue were gently transplanted under the left renal capsule of the recipients and, in the absence of abnormalities the abdominal cavity was closed. After procedure completion, the animals were placed in mini-cages at 37 °C until complete anesthetic recovery.

### Assessment of ovarian activity

Before receiving the ovarian fragments, the recipients had their estrous cycle monitored for 90 days and thus functioned as a control of themselves after the xenotransplantation.

Five days after the surgical procedure, vaginal washing was performed in the recipients to evaluate the estrous cycle every 12 hours for 60 days. The mice were gently restrained and washed intravaginally with a 20 μL micropipette filled with 0.9% saline and vaginal washes were evaluated under inverted light microscopy and without staining, in a 20x magnification. Classification of the estrous cycle phase through vaginal washing was based on previously established criteria (Cooper, 1993; Byers et al., 2012).

### Hormonal assay

The animals were intraperitoneally anesthetized with 2,2,2-tribromoethanol. The general anesthesia and its effectiveness were confirmed by the absence of foot reflex, cardiac puncture was then performed according to Hoff (2000). The total blood volume collected in the animals was approximately 1 mL. After this procedure the animals were euthanized. The death was confirmed by checking for the absence of vital signs such as heartbeat and respiratory movements. Blood from each animal was centrifuged at 2000g for 10 minutes at room temperature to obtain the plasma. The samples were then analyzed in duplicate for determining 17β-estradiol concentrations using amplified chemiluminescence using an immunoassay system (Vitros Eci/EciQ Immunodiagnostic System, Johnson and Johnson) according to the manufacturer’s instructions and following the methods of Bartoskova et al. (2014).

### Histological processing and evaluation

After death, the transplants were recovered and immediately immersed in 4% paraformaldehyde solution buffered with phosphate solution (pH 7.2) for 24 hours, subsequently dehydrated, diaphanized and embedded in paraffin, then sectioned semi-serially at a thickness of 5 μm, with one section mounted every 60 μm. Next, they were stained with hematoxylin-eosin (HE) and Masson’s trichrome (MT).

The ovarian tissue was assessed according the integrity of its parenchyma, quality and classification of preantral and antral follicles, as well as vascularization and aggregation of stromal cells. The follicles were classified according to morphology and number of granulosa cell layers surrounding the oocyte as primordial, primary, secondary and antral (Kumari et al., 2017). Larger antral follicles were aspirated during withdrawal.

### Stereological vascularization analysis

One image from each section of the xenotransplantation was randomly captured on 10X objective, totaling 10-12 photomicrographs. These were overlaid by a test system used to measure the volume of each fragment by the Cavallieri’s principle, as described by Noorafshan et al. (2013).

The volume of the xenotransplanted ovary fragment was determined using the following Formula 1: 

$$Volume\;=\;\sum p\;x\;a\left(p\right)\;x\;T$$ (1)

where Σp is the sum of points that overlaps the image; a(p) is the area associated with each point; and T is the distance between each section of the sample.

**Figure 1:**
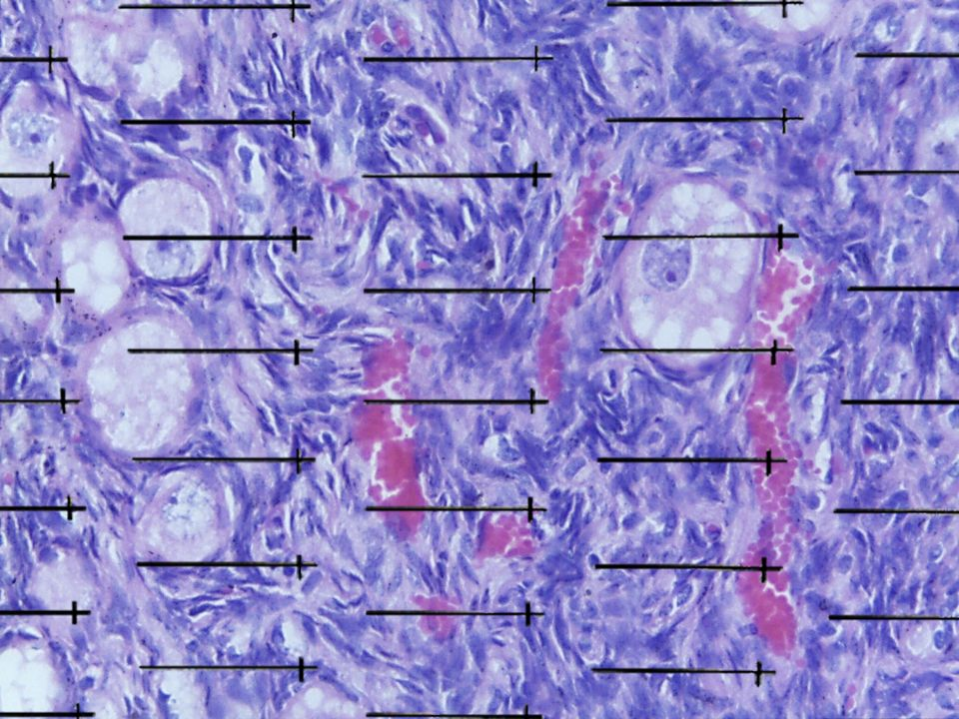
Ovary fragment of xenotransplanted goat fetus in the renal subcapsular region of mice withoverlap of the grid used to determine the volume surface density of the blood vessels of the fragment. Scale bar = 10 μm. HE staining.

Five fields from the same sections were randomly photographed in the 40X objective using a microscope coupled to a camera (LEICA DM500) to determine the volume density and area of blood vessels. A test system was superimposed on the images for quantification (Figure 1).

The volume density (Vv) of the vessels in the fragments of the two groups was calculated by the following Formula 2: 

$$Vv\;=\;\left(\Sigma p\left(Vessels\right)\right)/\left(\Sigma p\left(total\right)\right)$$ (2)

where Σp(Vessels) is the sum of points that touch the blood vessels; and Σp(total) is the total points of the test system. 

The area density (Sv) was measured by the Formula 3:

$$Sv\;=\;2I/\left(\Sigma p\;x\;I\left(p\right)\right)$$ (3)

where I corresponds to the number of line intersections in the vessels; I (p) is the length of the line; and Σp is the sum of the points that touch the xenotransplanted ovarian tissue. 

The total surface of the vessels (μm^2^) was determined by multiplying the Sv by the xenotransplanted fragment volume.

### Analysis of results

Data were tabulated and submitted to descriptive statistics. The chi-square test (χ^2^) was used for the data obtained in the analyzes of the follicular proportions. For comparing counting data (absolute values) between treatments, the data were previously submitted to assumptions (Kolmogorov-Smirnov, Shapiro-Wilk, Levene, Lilliefors) after verifying heteroscedasticity and non-Gaussian distribution, the samples were analyzed by the Kruskal-Wallis test followed by a Dunn post-hoc test. The software package used was the STATISTICA^®^ 8 program.

Mean duration of estrous cycle phases, biochemical data and hormone dosage were compared by ANOVA followed by the Tukey test. The development data of the transplanted tissue were put into percentage, with BioEstat 5.0 and Microsoft Office Excel 14.0 being used for this purpose.

Stereology data were analyzed using the Mann-Whitney test using Graph Prism version 3.05.

All comparisons were performed according to the experimental groups with probability level of 5%.

## Results

### Macroscopic findings

**Figure 2:**
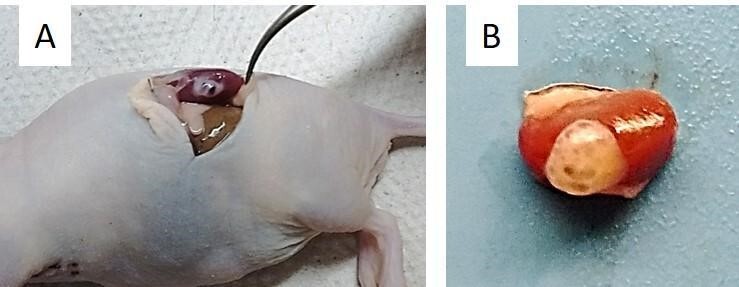
Fragment of ovarian cortex of goat fetus collected after 65 days of xenotransplantation immunosuppressed recipients (A), demonstrating ovarian development under renal capsule (B).

Transplanted tissue development was observed immediately after recovery, presenting normal appearance as well as several antral follicles on its surface. This observation was further confirmed by histological evaluation, thus confirming that there was 100% (n = 05/05) development of the transplanted fresh tissue, and 80% (n = 8/10) after cryopreservation and xenotransplantation in the females of both lineages: C57BL6 SCID and BALB/c NUDE (Figure 2). In the other 20% of the tissue after cryopreservation and xenotransplantation, only ovarian fibroblasts were observed.

### Microscopic findings

#### Vaginal lavage evaluation

**Figure 3:**
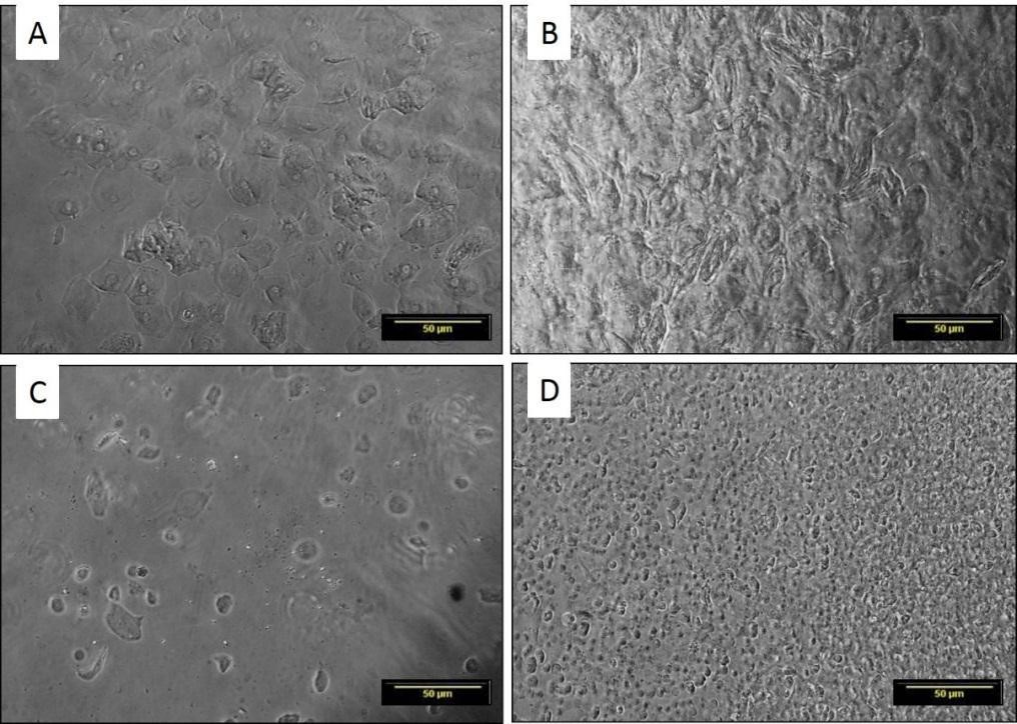
Cytological pattern of vaginal lavage of immunosuppressed mice after xenotransplantation. (A) proestrus - presence of rounded and nucleated epithelial cells (basal and parabasal); (B) estrus - keratinized cells in greater proportion; (C) metaestrus - reduction in the amount of keratinized cells, some leukocyte cells and appearance of some epithelial cells; (D) diestrous - small proportion of epithelial and keratinized cells and large amount of leukocyte cells. Scale bar: 50 μm. Increased by 20x.

Considering the mice lineages used, all recipients presented a return to ovarian activity rate with complete estrous cycle after fresh transplantation, and 80% (n = 8/10) after cryopreservation and xenotransplantation in the females of both lineages: C57BL6 SCID and BALB/c NUDE. The four phases of the estrous cycle were observed in all experimental animals (Figure 3), but some of these were more prolonged in the transplanted animals (Table 1, p <0.05). The onset of this activity after xenotransplantation was on average 13 ± 6 days from the FX group and 17 ± 9 days from the CX group in the C57BL6 SCID females, and 4 ± 2 days from the FX group and 10 ± 5 days from the CX group in the BALB/c NUDE females, observing irregularity in the estrous cycle of transplanted animals.

**Table 1:** Mean and standard deviation (hours) of the phase durations of the first estrous cycle of the different experimental groups.

**Experimental groups**		**Estrous cycle stages**			
		**Proestrus**	**Estrus**	**Metaestrus**	**Diestrus**
	Control	12.0 ± 0.7^aB^	96.0 ± 1.9^aA^	12.0 ± 0.0^aB^	48.0 ±1.2^bB^
C57BL6 SCID	Fresh	24.0 ± 1.0 ^aB^	32.0 ± 0.0^bA^	12.0 ± 0.0^aA^	160.0 ± 12.3^aA^
	Cryo	36.0 ± 1.7^aA^	68.0 ± 12.5^aA^	12.0 ± 0.0^aA^	172.0 ± 2.5^aA^
					
	Control	72.0 ± 0.9^aA^	48.0 ± 0.9^bB^	24.0 ± 0.8^aA^	72.0 ± 1.1^bA^
BALB/c NUDE	Fresh	40.0 ± 1.5^bA^	36.0 ± 0.0^bA^	12.0 ± 0.0^bA^	120.0 ± 3.0^aB^
	Cryo	25.0 ± 1.1^bA^	60.0 ± 4.2^aA^	12.0 ± 0.0^bA^	111.0 ± 4.3^aB^

Lower case letters indicate comparison between treatments within the same lineage. Upper case letters indicate comparison between the same treatment among different transplanted lineages.

### Morphology of preantral and antral follicles

**Figure 4:**
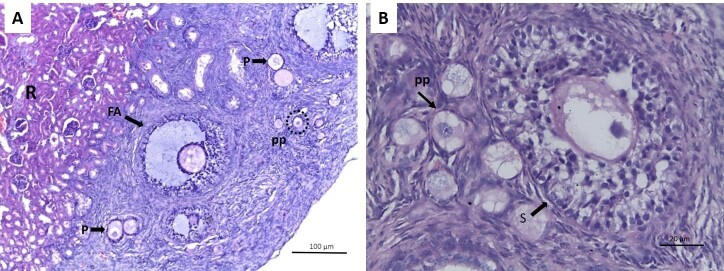
Photomicrography of mongrel goat fetus ovary after ovarian xenotransplantation in immunosuppressed C56BL6 SCID receptors. (A) primordial follicles (pp), antral follicle (FA), primary follicle (P), renal region of the recipient (R). Increase by 10x; (B) secondary follicle (S), primordial follicle (pp), Hematoxylin-Eosin staining (HE). Increased by 40x.

Follicles were observed at all development stages (Figure 4). Therefore, both lineages and the chosen recipient site allowed follicular development in the antral phase after both fresh and cryopreservation transplantation. Upon observation the atresic follicles presented degeneration of the oocytes accompanied by rupture of granulosa cell layers and pycnotic nuclei.

### Proportions of follicular stages

Complete follicular development was observed after fresh and cryopreserved ovarian xenotransplantation in both lineages. When compared to control, a decrease of preantral follicles of both treatments were observed in both lineages (Table 2, p <0.05).

**Table 2:** Follicular proportion (%) of fetal ovary xenotransplantation under the renal capsule of C56BL6 SCID and BALB-C NUDE mice.

**Treatments**		**Total follicles**	**FOLLICULAR CATEGORY**				
			**Primordial**	**Primary**	**Secondary**	**Initial Antral**	**Antral**
Fresh Ovary	FO	n = 1288	88.3 ± 2.4 ^a^	11.7 ± 2.4 ^b^	0 ± 0.0 ^b^	0 ± 0.0 ^c^	0 ± 0.0 ^c^
Fresh	SCID	n = 2757	47.7 ± 5.3 ^bA^	27.6 ± 6.4 ^bA^	13.3 ± 4.7% ^aA^	7.1 ± 1.9 ^aA^	4.2 ± 0.5 ^aA^
	NUDE	n = 2599	46.6 ± 7.1 ^bA^	30.5 ± 5.3 ^aA^	16.7 ± 3.4 ^aA^	3.7 ± 0.8 ^bA^	2.4 ± 0.7 ^bB^
Cryo	SCID	n = 200	46.6 ± 10 ^bA^	25.1 ± 5% ^bA^	22.9 ± 7 ^aA^	3.8 ± 4.0 ^bA^	1.5 ± 3.0 ^bB^
	NUDE	n = 90	38.7 ± 33 ^bA^	39.5 ± 26 ^aA^	12.4 ± 10 ^aA^	4.4 ± 6.0 ^abA^	5.1 ± 8.0 ^aA^

Lower case letters compare the control group with each treatment. Uppercase letters compare the treatments between lines (i.e. fresh SCID x cryo SCID).

### Vascular supply for transplanted tissue

**Figure 5:**
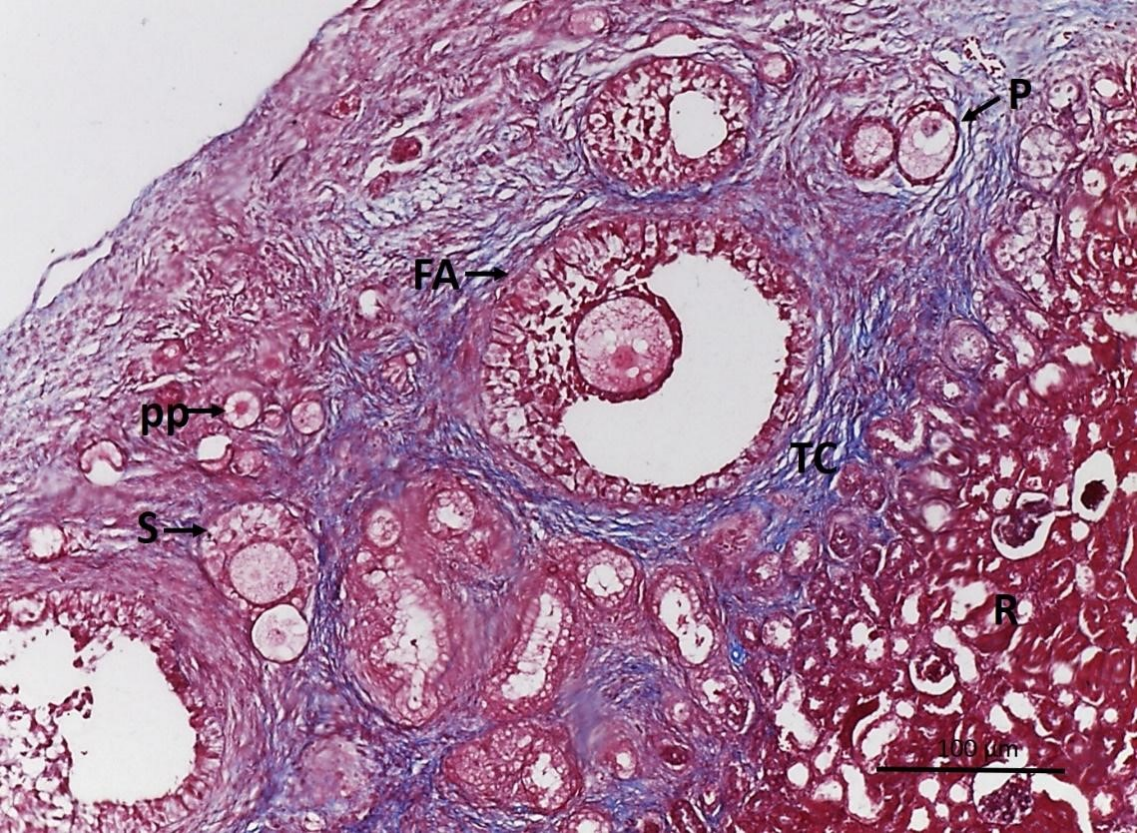
Photomicrography of goat fetus ovary after ovarian xenotransplantation in immunosuppressed BALB-C NUDE receptors. Antral follicle (FA), secondary follicle (S), primary follicle (P), primordial follicle (pp). Note the cortical tissue integration of the ovary and the renal parenchyma of the operated recipient (R) and connective tissue (TC), Masson’s trichrome staining (MT). Increased by 10x.

Sixty-five (65) days after the xenotransplantation, the vascular supply provided by the recipient’s kidney to the ovarian cortex fragments of the donors was considered morphologically similar to that existing in the in situ ovary of the donors, with revascularization occurring in 100% of the fresh transplants and in 80% of the cryopreserved transplants of both recipient lineages (C57BL6 SCID and BALB/c NUDE), with the same proportions being found in the return of the ovarian activity of these animals. Blood vessels were histologically observed in the recovered transplants (Figure 5).

### Stereological study of blood vessels

After counting all the points and intersections of the test system, it was found that the volume of the fragments, the volume density and the surface density presented a difference (p <0.01) between the treatments of the Balb-c NUDE lineage, as shown in Table 3.

**Table 3:** Effect of vitrification on ovarian xenotransplant volume, volume density (Vv), vessel area (Sv).

**Lineage**	**Treatments**	**Volume (mm^3^)**	**Vv (%)**	**Sv (µm^-1^)**
C57BL6 SCID	FX	34825.7^a^	12^a^	36.18^a^
	CX	13038.7^a^	5.71^a^	5.93^a^
BALB/c NUDE	FX	35692.2^a^	16^a^	68.39^a^
	CX	7839.98^b^	3.33^b^	3.46^b^

Lowercase letters compare treatments of the same lineage. FX: Fresh Xenotransplantation; CX: Xenotransplantation after cryopreservation.

### Hormonal assessment

Estradiol hormone assays from the recipient mice were performed based at the end time of the experiment and, in C57BL6 SCID recipients, the levels ranged from 37.59 to 56.40 pg/ml submitted to FX group, whereas it ranged from 35.26 to 56.60 pg/ml in CX group. In the FX group Balb/c NUDE recipients, the values ranged from 34.98 to 50.21 pg/ml, and those from the CX group ranged from 40.60 to 51.23 pg/ml (Table 4, p > 0.05).

**Table 4:** Estradiol hormone dosage after ovary xenotransplantation of goat fetus under the renal capsule of C56BL6 SCID and BALB-C NUDE mice.

**Lineage**	**Treatments**	**Dosage (pg/mL)ns**
C57BL6 SCID	Fresh	48.04 ± 8.00
	Cryo	42.48 ± 5.93
BALB/c NUDE	Fresh	47.07± 8.37
	Cryo	45.23 ± 4.06

ns There were no differences between the performed comparisons (p > 0.05).

## Discussion

The present study provides relevant information for rescuing preantral follicles in ovaries of goat fetuses after xenotransplantation. The use of animal germplasm is growing on the part of researchers and laboratories interested to develop economically and ecologically valuable animals. Some individuals may find themselves in situations of vulnerability such as prey or hunting due to anthropic action. Investigations with xenotransplantations were performed in domestic mammals (Gosden et al., 1994; Barros et al., 2014; Tahaei et al., 2015; Santos et al., 2016) and wild ones (Wiedemann et al., 2012; Praxedes et al., 2018).

The return of ovarian activity in the recipients and the presence of a high number of primordial follicles as well as antral follicles after xenotransplantation confirmed the hypothesis that this biotechnique represents a promising tool for recovering ovarian function and to elucidate revascularization mechanisms in fresh or cryopreserved tissues (Youm et al., 2014). However, preservation of fertility using ovarian tissue transplants is still in the experimental phase (Lee et al., 2015) and is slowly being adapted not only for animals (Ayuandari et al., 2016). Already being widely used in humans (Mofarahe et al., 2020; Mohammadi et al., 2020).

Tissue adhesion and the presence of vascularization were macroscopically observed in all treatments, that demonstrated the efficacy of growth and revascularization during the evaluated transplant time. Reperfusion played an important role in preventing follicle depletion during the first few days to one week after transplantation (Soleimani et al., 2011). During this phase, ischemia and hypoxia occurs until the formation of new blood vessels within both the transplantation and the surrounding tissue (Van Eyck et al., 2010), thus, with complete revascularization the oxygen and nutrients of the surrounding tissue are diffused into the transplanted tissue, restoring it.

The efficiency of this blood reperfusion had a reflex in the estrus observation in the recipients. Determining the estrous cycle is of paramount importance in xenotransplantation procedures, since they serve as an indirect evidence of the hormonal changes resulting from readapting the transplanted tissues and are reflected in the cycle. In this study, irregularities in the estrous cycle of the animals after xenotransplantation were observed in both treatments when compared to the control, with longer phases (in the estrus and diestrus of the Balb/c NUDE and in the estrus in the C57BL6 SCID mice recipients) and other shorter phases (proestrus and metaestrus of the Balb/c NUDE recipients and in the diestrus of the C57BL6 SCID recipients). It is believed that the physiological state of the recipients may influence transplantation and/or follicular survival. In some ovarian transplantation studies, bilateral ovariectomized females were performed due the consecutive release of higher concentrations of gonadotrophins after ovariectomy (Gosden et al., 1994; Wolvekamp et al., 2001). This initial hormonal decompensation after ovariectomy possibly caused irregularities in the estrous cycle after transplantation, since the hypothalamic-pituitary-gonadal (HPG) axis of the recipients was not yet adapted to the newly incorporated gonad.

In relation to follicular development, many culture systems were developed in the last decades for improvement of primordial follicle activation and preantral follicle growth by autotransplantation (Santos et al., 2009) and in vitro culture until they are able to use in vitro fertilization in goats (Magalhães et al., 2011). Similar results were observed here, once the development of the follicles until the antral stage was verified. During fetal life, primordial follicles are activated around the 73^rd^ day post conception and antral follicles are shown from the beginning of the last third of gestational life with small changes in the folliculogenesis kinetics in the preantral phase (Bezerra et al., 1998). The possibility of development and differentiation prior to interrelating the HPG axis and the early ovarian responsiveness to gonadotrophins in goat fetuses were recently considered in fetus of this species (Kumari et al., 2017), with possibilities mainly being plausible when the gonadotrophin source comes from another species like that presented in this study.

The kinetics of follicular development were evidenced when follicular proportions were observed between the different treatments and lineages, with a follicular right shift being observed. A phenomenon characterized by a decrease in the number of primordial follicles and a gradual increase of primary, secondary and antral follicles due to follicle activation and follicular growth after xenotransplantation (Santos et al., 2016), showing that in vivo culture by fetal goat ovary xenotransplantation was efficient and allowed for normal follicular development.

The number of viable follicles was not different between groups, the only exception was in case of antral follicles between FX and CX group of the C57BL6SCID lineage. It is known that cryogenic injuries can affect follicular development, reducing cellular respiration, increasing DNA degeneration and releasing intracellular material (Laschke et al., 2003). However, the current consensus is that ischemia prior to transplantation can lead to damage induced by cryopreservation, which is the main cause of follicular loss (González et al., 2012). In this study, there was a reduction in follicular density after vitrification, but this did not prevent the existing follicles in both mice lineages from developing to antral follicles, which were probably favored by less competition for nutrients.

The renal subcapsular region offers several advantages for maintaining transplanted tissue as a recipient site: the same embryological origin of the reproductive tract and the potential vascular support around 20 to 25% of the cardiac output (Christie and Bjorling, 2003). For this study, the renal subcapsular region was chosen as the recipient site of the ovarian tissues by this site to prevent the extrusion of the ovarian tissue into the abdominal cavity. Another reason for choosing this site was that the renal parenchyma provides better vascularization, since it avoids extending the distance of the transplanted tissue from the blood supply, thus enabling faster follicular activation after transplantation (Aerts et al., 2010).

The data presented by the stereological evaluation showed that the volume of the fragments, the volume density and the vessel area of the Balb/c NUDE lineage was lower in the transplanted ovarian tissue after cryopreservation when compared to the fresh transplanted tissue (p <0.01). Better results were found in the C57BL6SCID lineage, with no difference (p> 0.05) between treatments, demonstrating that these mice may be a better recipient for transplanted tissues after cryopreservation compared to Balb/c NUDE. Stereology studies were essential to demonstrate the xenotransplantation efficacy for revascularizing the fragments in the different treatments. The findings on the quantification of ovarian follicles after transplantation associated with vascularization conferred the best performance for C57BL6 SCID lineage recipients.

Estradiol levels in the mice after goat fetus ovarian xenotransplantation was found to be within the range expected for the species and lineage (Ingberg et al., 2012). After an ischemic, the fetal ovary was able to revascularize under the renal capsule and possibly induced reactivation of the hypothalamus-hypophysis-gonadal axis of the recipients, and thereby allowing the massive production of 17β-estradiol by the implanted fragment which helped in follicular development. This hormone is considered the most potent natural estrogen produced by the ovaries in most species. It is produced predominantly by the aromatization of testosterone by granulosa cells (GC) of the ovarian follicle. In the follicular phase of the cycle, the increased rate of secretion of FSH induces expression of the aromatase enzyme in GC. As a result, estrogen production in the follicle increases rapidly. The rising of FSH serum concentration leads also to an accelerated growth of the follicle largely due to the rapid secretion and accumulation of follicular fluid (FF). The FF is also formed by GC (Bentov et al., 2016).

## Conclusion

The xenotransplantation of goat fetal ovaries, freshly or after their cryopreservation allowed, without the use of hormonal protocols, the survival, growth and development of antral follicles.

Considering the importance of transplantation studies for preserving fertility and shortening capacity between generations, the xenotransplantation technique applied in this work may provide an in vivo model to study population and follicle development from fetal ovaries in several species, including wild goats. In addition, in spite of reducing the number of available ovarian follicles, ovarian fetal tissue xenotransplantation still provides satisfactory follicular development and restores endocrine function in the recipients, in which all the estrous cycle phases were observed.
